# Pulmonary alveolar proteinosis: An autoimmune disease lacking an HLA association

**DOI:** 10.1371/journal.pone.0213179

**Published:** 2019-03-07

**Authors:** Kirsten Anderson, Brenna Carey, Allison Martin, Christina Roark, Claudia Chalk, Marchele Nowell-Bostic, Brian Freed, Michael Aubrey, Bruce Trapnell, Andrew Fontenot

**Affiliations:** 1 University of Colorado Anschutz Medical Campus, Department of Medicine, Aurora, CO, United States of America; 2 ClinImmune Labs Aurora, CO, United States of America; 3 Cincinatti Children’s Hospital Medical Center (CCHMC) Cincinnati, OH, United States of America; Universitatsklinikum Freiburg, GERMANY

## Abstract

Pulmonary alveolar proteinosis (PAP) is a rare lung disease characterized by the accumulation of pulmonary surfactant in alveolar macrophages and alveoli, resulting in respiratory impairment and an increased risk of opportunistic infections. Autoimmune PAP is an autoimmune lung disease that is caused by autoantibodies directed against granulocyte-macrophage colony-stimulating factor (GM-CSF). A shared feature among many autoimmune diseases is a distinct genetic association to *HLA* alleles. In the present study, we HLA-typed patients with autoimmune PAP to determine if this disease had any *HLA* association. We analyzed amino acid and allele associations for *HLA-A*, *B*, *C*, *DRB1*, *DQB1*, *DPB1*, *DRB3*, *DRB4* and *DRB5* in 41 autoimmune PAP patients compared to 1000 ethnic-matched controls and did not find any *HLA* association with autoimmune PAP. Collectively, these data may suggest the absence of a genetic association to the *HLA* in the development of autoimmune PAP.

## Introduction

Pulmonary alveolar proteinosis (PAP) is a rare syndrome comprising a heterogeneous group of diseases characterized by the accumulation of pulmonary surfactant in alveolar macrophages and the alveolar space [1, 2]. Eventually, surfactant accumulation results in respiratory impairment and/or failure as well as an increased risk of opportunistic infections [3]. This syndrome occurs in individuals from ages 8 to 90 years, but it is most common in male smokers in the third to fourth decade [4, 5]. The catabolism of pulmonary surfactant in alveolar macrophages is controlled by granulocyte-macrophage-colony stimulating factor (GM-CSF) [6]. GM-CSF is a cytokine that modulates the survival, differentiation, proliferation, and priming of myeloid cells [7]. GM-CSF signaling can be disrupted by mutations in the GM-CSF gene [8, 9] or its receptors [10–13], as well as by neutralizing autoantibodies [2, 14, 15]. In this regard, autoimmune PAP is the disease that results from autoantibodies directed against GM-CSF, and the identification of neutralizing, polyclonal anti-GM-CSF autoantibodies in autoimmune PAP is an essential component of the disease diagnosis.

In most autoimmune diseases, CD4^+^ T cells are required to assist B cells in isotype switching, which is necessary for the generation of autoantibodies such as those found in autoimmune PAP patients. Few studies have investigated the role of the adaptive immune response, and no studies to date have investigated the association between the genetic susceptibility to autoimmune PAP and *HLA*. In the vast majority of immune-mediated diseases, genetic susceptibility has been most strongly associated with the *HLA* region on the short arm of chromosome 6 [16]. For example, the major genetic contribution to rheumatoid arthritis (RA) involves *DRB1* alleles such as DRB1*04:*01* and *04:*04* [17], whereas *HLA-DQ* alleles, especially *DQB1*:*02*:*01* and *DQB1*:*03*:*02*, provide the major genetic contribution to type 1 diabetes (T1D) [16]. In the case of chronic beryllium disease, genetic susceptibility is strongly linked to *HLA-DPB1* alleles possessing a glutamic acid at position 69 of the β-chain [18]. Thus, the impetus for this study was to identify to *HLA* alleles that confer susceptibility to the generation of autoimmune PAP.

## Methods

### Patients and controls

We collected DNA from 41 Caucasian subjects with autoimmune PAP defined by a serum level of GM-CSF autoantibody greater than 5.6 μg/mL. These patients were recruited from the Translational Pulmonary Science Center (TPSC) at Cincinnati Children’s Hospital Medical Center (CCHMC), the University of Cincinnati Medical Center, and other Rare Lung Disease Network (RLDN) centers/affiliates. The Cincinnati Children's Hospital Medical Center Office of Research Compliance and Regulatory Affairs issued for this study IRB # 2011–0147. Written consent was obtained from all patients enrolled in the study. The patients were all Caucasians with a positive GM-CSF autoantibody test [19], with a mean age of 44.3+/-6.7 years (+/-SEM), and comprised of 40.9% female and 59.1% male, from various regions in the United States (84.1%), Canada (2.3%), Turkey (13.6%) Subjects and demographic information are available in S1 Table.

High resolution molecular typing for *HLA-A*, *B*, *C*, *DRB1*, *DPB1*, *DQB1*, *DRB3*, *DRB4* and *DRB5* was performed at ClinImmune Laboratories at the University of Colorado Anschutz Medical Campus. 1,000 control subjects for *HLA* analysis were acquired from the National Marrow Donor Program (NMDP) Full HLA types for patients and controls are published in S2 Table. For validation of small sample sizes in HLA Epitope Analysis Program (HEAP), we used data from patients with RA (patient data from IHWG) and T1D (data from the T1D Genetics Consortium).

### HLA analysis and statistics

The number of patients carrying at least one copy of each allele was counted and compared with the number of controls carrying at least one copy of the same allele. The significance of each was calculated using a Chi-square test or Fisher’s exact test as appropriate depending on the number of subjects in each contingency table. The resulting p-values were corrected for multiple comparisons using the false discovery rate method [20]. We additionally performed an equivalence test for all alleles using R software version 3.4.4 and the package TOSTER (https://cran.r-project.org/web/packages/TOSTER/index.html).

HLA amino acid association analysis was performed using the R software package version 2.6.1. Combinations of 1–4 polymorphic amino acids at positions 8–93 of HLA-DRB1, DPB1 and DQB1, as well as combinations of up to 4 amino acids at positions 2–192 of HLA-A, B and C were analyzed for association with PAP. Chi-square or Fisher’s exact tests were calculated as appropriate, and the P-values were adjusted for multiple comparisons.

### HEAP validation for small sample sizes

In small sample sizes, allele association studies are likely to miss true positive associations due to polymorphisms in the *HLA* region. Rare alleles that may be truly disease susceptible alleles cannot reach statistical significance due to their low frequency in both patient and control groups. Amino acid association tests, on the other hand, benefit small studies by identifying shared features between disparate alleles, including rare alleles.

To test whether we could detect a true positive HLA association with small patient numbers, we analyzed known HLA amino acid associations from RA and T1D. RA is strongly associated with alleles of DRB1*04, which in single amino acid analysis can be identified by the presence of histidine at position 13 [20]. T1D has strong single amino acid associations in both HLA-DRB1 (histidine at position 13) and HLA-DQB1 (alanine at position 57) [21]. In T1D, the DQB1 association is stronger than the association in DRB1 [21]. We determined whether we could correctly identify the known HLA amino acid associations in RA and T1D through random sampling of 50, 40, 30 and 20 patients. We performed each random sampling ten times for each level of patient numbers and with 1,000 NMDP controls each. With these data, we determined how frequently HEAP could identify the known amino acid association for RA and T1D. Statistics were performed as described above for PAP association.

## Results

### *HLA* Allele frequency and amino acid polymorphism in autoimmune PAP

We collected DNA from 47 Caucasian patients with autoimmune PAP from Cincinnati Children’s Medical Center as part of the RLDN. We performed high resolution typing of all classical loci of HLA class I and class II. We analyzed both allele frequencies and amino acid frequencies between patients and 1000 ethnic-matched controls provided by the NMDP. To identify potential shared amino acid sequences, we used HEAP, which compares all polymorphic amino acids in a locus [21–23]. We examined potential HLA shared amino acids from 1 to 4 non-contiguous amino acids and analyzed a total of 5,699,818 possible amino acid combinations. However, we did not find any significant associations at the amino acid level (S9–S32 Tables). We also calculated allele frequencies for all loci and performed a chi-square analysis between patients and controls. In our control and patient populations, we identified 203 different alleles in *HLA-A*, *B*, *C*, *DRB1*, *DPB1* and *DQB1* (Tables 1–6), and none were significantly associated with autoimmune PAP. Many of the alleles tested were rare (<1% in both patient and control groups) and were not found to be associated with PAP. For full allele results, see S3–S8 Tables.

**Table 1:** HLA-A alleles in patients and controls.

Allele	Patients (%)	Controls (%)	p—value	p—adjusted
01:01	9 (30.0)	281 (28.1)	0.84	1
02:01	12 (40.0)	487 (48.7)	0.35	1
02:05	1 (3.3)	16 (1.6)	0.40	1
02:06	1 (3.3)	1 (0.1)	0.06	1
03:01	9 (30.0)	282 (28.2)	0.84	1
11:01	3 (10.0)	112 (11.2)	1	1
23:01	1 (3.3)	45 (4.5)	1	1
24:02	6 (20.0)	155 (15.5)	0.45	1
25:01	2 (6.7)	44 (4.4)	0.39	1
26:01	3 (10.0)	61 (6.1)	0.43	1
29:01	1 (3.3)	10 (1.0)	0.28	1
29:02	5 (16.7)	60 (6.0)	0.04	1
30:01	0 (0.0)	33 (3.3)	0.62	1
30:02	2 (6.7)	12 (1.2)	0.06	1
31:01	0 (0.0)	47 (4.7)	0.39	1
32:01	1 (3.3)	73 (7.3)	0.72	1
33:01	0 (0.0)	17 (1.7)	1	1
68:01	1 (3.3)	53 (5.3)	1	1
68:02	1 (3.3)	14 (1.4)	0.36	1

**Table 2:** HLA-B alleles in patients and controls.

Allele	Patients (%)	Controls (%)	*p*—value	*p*—adjusted
07:02	10 (33.3)	264 (26.4)	0.40	1
07:05	1 (3.3)	4 (0.4)	0.14	1
08:01	6 (20.0)	190 (19.0)	0.82	1
13:02	0 (0.0)	65 (6.5)	0.25	1
14:01	1 (3.3)	14 (1.4)	0.36	1
14:02	2 (6.7)	41 (4.1)	0.36	1
15:01	3 (10.0)	118 (11.8)	1	1
18:01	2 (6.7)	84 (8.4)	1	1
27:02	0 (0.0)	18 (1.8)	1	1
27:05	2 (6.7)	65 (6.5)	1	1
35:01	4 (13.3)	133 (13.3)	1	1
35:02	1 (3.3)	20 (2.0)	0.47	1
35:03	2 (6.7)	30 (3.0)	0.24	1
35:08	1 (3.3)	6 (0.6)	0.19	1
37:01	2 (6.7)	20 (2.0)	0.13	1
38:01	1 (3.3)	33 (3.3)	1	1
39:01	0 (0.0)	34 (3.4)	0.62	1
40:01	2 (6.7)	88 (8.8)	1	1
40:02	0 (0.0)	22 (2.2)	1	1
44:02	3 (10.0)	173 (17.3)	0.46	1
44:03	6 (20.0)	105 (10.5)	0.13	1
44:05	1 (3.3)	12 (1.2)	0.32	1
49:01	1 (3.3)	44 (4.4)	1	1
50:01	2 (6.7)	20 (2.0)	0.13	1
51:01	1 (3.3)	85 (8.5)	0.51	1
51:08	1 (3.3)	4 (0.4)	0.14	1
52:01	2 (6.7)	23 (2.3)	0.16	1
55:01	1 (3.3)	42 (4.2)	1	1
56:01	0 (0.0)	14 (1.4)	1	1
57:01	2 (6.7)	78 (7.8)	1	1
58:01	0 (0.0)	15 (1.5)	1	1

**Table 3:** HLA-C alleles in patients and controls.

Allele	Patients (%)	Controls (%)	*p*—value	*p*—adjusted
01:02	3 (10.0)	6 (6.0)	0.45	1
02:02	1 (3.3)	9.7 (9.7)	0.36	1
03:03	6 (20.0)	10.4 (10.4)	0.15	1
03:04	4 (13.3)	13.4 (13.4)	1	1
04:01	8 (26.7)	22.7 (22.7)	0.83	1
05:01	4 (13.3)	16.3 (16.3)	0.64	1
06:02	4 (13.3)	18.9 (18.9)	0.49	1
07:01	7 (23.3)	27.6 (27.6)	0.55	1
07:02	13 (43.3)	27.9 (27.9)	0.17	1
07:04	0 (0.0)	3 (3.0)	0.62	1
08:02	3 (10.0)	5.4 (5.4)	0.42	1
12:02	1 (3.3)	2.2 (2.2)	0.53	1
12:03	3 (10.0)	9.8 (9.8)	1	1
14:02	0 (0.0)	2.4 (2.4)	1	1
15:02	0 (0.0)	4.4 (4.4)	0.39	1
15:05	1 (3.3)	0.5 (0.5)	0.18	1
16:01	4 (13.3)	6.4 (6.4)	0.27	1
16:02	1 (3.3)	0.9 (0.9)	0.28	1
16:04	1 (3.3)	0.3 (0.3)	0.12	1
17:01	0 (0.0)	1.5 (1.5)	1	1

**Table 4:** HLA-DRB1 alleles in patients and controls.

Allele	Patients (%)	Controls (%)	p—value	p—adjusted
01:01	7 (17.9)	169 (16.9)	0.83	1
01:02	1 (2.6)	21 (2.1)	0.57	1
01:03	0 (0.0)	16 (1.6)	1	1
03:01	4 (10.3)	210 (21.0)	0.15	1
04:01	1 (2.6)	153 (15.3)	0.02	1
04:02	2 (5.1)	20 (2.0)	0.20	1
04:03	1 (2.6)	15 (1.5)	0.46	1
04:04	0 (0.0)	60 (6.0)	0.16	1
04:05	0 (0.0)	10 (1.0)	1	1
04:07	1 (2.6)	12 (1.2)	0.39	1
07:01	14 (35.9)	260 (26.0)	0.17	1
08:01	2 (5.1)	50 (5.0)	1	1
08:02	1 (2.6)	2 (0.2)	0.11	1
08:03	1 (2.6)	5 (0.5)	0.21	1
09:01	0 (0.0)	15 (1.5)	1	1
10:01	4 (10.3)	12 (1.2)	0.00	0.36
11:01	4 (10.3)	128 (12.8)	0.81	1
11:02	1 (2.6)	8 (0.8)	0.29	1
11:03	3 (7.7)	21 (2.1)	0.06	1
11:04	4 (10.3)	57 (5.7)	0.28	1
12:01	0 (0.0)	28 (2.8)	0.62	1
13:01	3 (7.7)	138 (13.8)	0.35	1
13:02	4 (10.3)	88 (8.8)	0.77	1
13:03	1 (2.6)	19 (1.9)	0.54	1
14:01	3 (7.7)	53 (5.3)	0.46	1
14:06	1 (2.6)	0 (0.0)	0.04	1
15:01	11 (28.2)	269 (26.9)	0.86	1
15:02	0 (0.0)	19 (1.9)	1	1
16:01	2 (5.1)	40 (4.0)	0.67	1

**Table 5:** HLA-DPB1 alleles in patients and controls.

Allele	Patients (%)	Controls (%)	p—value	p—adjusted
01:01	6 (15.0)	99 (9.9)	0.28	1
02:01	8 (20.0)	261 (26.1)	0.39	1
02:02	1 (2.5)	11 (1.1)	0.38	1
03:01	6 (15.0)	206 (20.6)	0.55	1
04:01	21 (52.5)	675 (67.5)	0.05	1
04:02	17 (42.5)	227 (22.7)	0.01	0.71
05:01	2 (5.0)	27 (2.7)	0.31	1
06:01	1 (2.5)	41 (4.1)	1	1
09:01	1 (2.5)	10 (1.0)	0.35	1
10:01	1 (2.5)	29 (2.9)	1	1
11:01	3 (7.5)	30 (3.0)	0.13	1
13:01	1 (2.5)	36 (3.6)	1	1
14:01	3 (7.5)	24 (2.4)	0.08	1
15:01	0 (0.0)	11 (1.1)	1	1
16:01	0 (0.0)	14 (1.4)	1	1
17:01	1 (2.5)	31 (3.1)	1	1
19:01	1 (2.5)	18 (1.8)	0.53	1
23:01	0 (0.0)	15 (1.5)	1	1

**Table 6:** HLA-DQB1 alleles in patients and controls.

Allele	Patients (%)	Controls (%)	*p*—value	*p*—adjusted
02:01	4 (10.0)	381 (38.1)	0.47	1
03:01	13 (32.5)	343 (34.3)	0.81	1
03:02	5 (12.5)	167 (16.7)	0.66	1
03:03	6 (15.0)	91 (9.1)	0.26	1
04:02	4 (10.0)	60 (6.0)	0.30	1
05:01	12 (30.0)	210 (21.0)	0.17	1
05:02	2 (5.0)	47 (4.7)	0.71	1
05:03	3 (7.5)	53 (5.3)	0.47	1
06:01	0 (0.0)	20 (2.0)	1	1
06:02	12 (30.0)	267 (26.7)	0.64	1
06:03	3 (7.5)	140 (14.0)	0.35	1
06:04	5 (12.5)	82 (8.2)	0.37	1
06:09	0 (0.0)	11 (1.1)	1	1

*DRB3*, *DRB4* and *DRB5* are three protein-coding loci in tight linkage disequilibrium with *DRB1*. These alleles pair with DRα, are expressed on the cell surface, and present peptides to T cells as any other class II allele. We imputed these alleles based on linkage analysis and examined the frequencies of these loci for potential association with PAP; however, no associations were identified (Table 7). Collectively, with our cohort, our data show that autoimmune PAP is unlikely to have an *HLA* association that can explain disease development.

**Table 7:** Alleles in linkage with DRB1 in patients and controls.

Allele	Patients (%)	Controls (%)	*p*—value	Odds Ratio
DRB3	25 (60.1)	632 (63.2)	0.869	0.91
DRB4	20 (48.7)	496 (49.6)	0.874	1.07
DRB5	10 (24.4)	324 (32.4)	0.311	0.67

Based on DRB1 types we determined the presence of a DR locus in linkage disequilibrium with DRB1 that may also contribute to disease. We calculated the odds-ratio for each additional locus and performed a chi-square analysis.

Given the small sample size, we performed a test of equivalence. In traditional t-tests, the null hypothesis is that the means in two groups are not different. Our initial analysis suggested that we cannot reject this null hypothesis. However, this does not demonstrate unequivocally that the groups are the same. In an equivalence test, the null hypothesis is that the two groups are different. A significant p-value in an equivalence test would allow us to reject this null hypothesis and indicates that the means between our two groups are equivalent. An equivalence test was performed for all alleles, and for each one we obtain a highly significant p-value, suggesting that there is no difference between the frequencies of *HLA* alleles between patients and controls. The maximum p-value we observed over all alleles tested was 4.23 x 10^−12^.

### Adequacy of sample size

**Fig 1:**
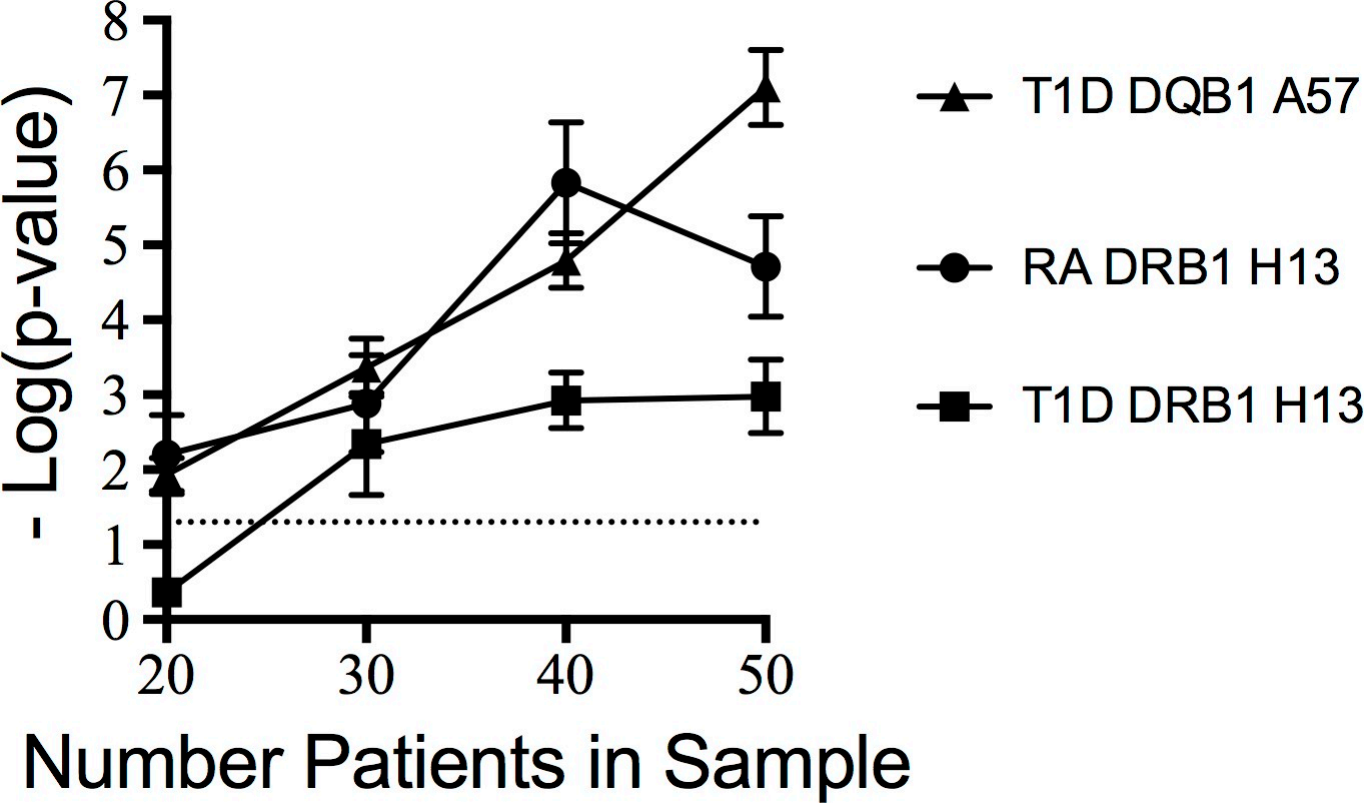
HEAP limits of detection by patient number. Identification of significant p-values with known disease associated epitopes by number of patients in the sample. Y-axis shows–Log(p-value) from the epitope analysis, X-axis shows the number of patient samples included in each analysis. For highly significant HLA associations, HEAP can consistently identify association with as few as 20 patients.

Due to the small number of autoimmune PAP subjects enrolled in the present study, we queried whether an association between HLA and similar numbers of subjects with diseases of known HLA associations (e.g., T1D and RA) could be detected. As shown in Fig 1, we found that HEAP could consistently identify a strong HLA association with as few as 20 patients in both T1D and RA. Thus, our findings suggest that our sample size of 47 should be adequate to detect an HLA association in autoimmune PAP, if a strong association exists.

## Discussion

Based on the autoimmune nature of PAP, we hypothesized that a link existed between this disease and *HLA*. However, our study suggests that no such link exists. It remains possible that the lack of an association was due to the small sample size (47 patients). However, we did not note any trends towards association. As discussed above, our sample size should be sufficient to identify a strong positive association. However, it may not be large enough to definitively conclude the absence of an association. Previous studies in our laboratory have conclusively demonstrated the lack of an *HLA* association with as few as 150 patients [22]. However, it should be noted that our methodology can identify a strong positive *HLA* association with only 50 patients [21]. Our HEAP analysis consistently identified a strong, single amino acid HLA association in T1D and RA with as few as 20 subjects. Collectively, these data suggest that our sample size should be capable of detecting an association if one were present. However, due to sample size considerations, we were forced to limit our study to Caucasian patients. A larger study with a more diverse population would allow a more conclusive assessment and to include other ethnic minorities who represent more diverse *HLA* alleles.

*HLA*-linked autoimmune diseases generally have a T cell-mediated component. Our initial hypothesis was based on the finding of an increased number of CD4^+^ and CD8^+^ T cells in the bronchoalveolar lavage of patients with PAP [24]. This suggests HLA involvement in presenting self-antigen to T cells which may then lead to CD4^+^ T cells helping B cells to class-switch. This would be consistent with our observations that antibodies against GM-CSF in autoimmune PAP patients are IgG [14]. However, it is possible that the increased number of T cells observed in the lungs of PAP patients was secondary to infection and can still account for the class-switched antibodies against GM-CSF. The absence of an *HLA* association in this cohort of autoimmune PAP subjects raises the possibility that the T cell alveolitis may be a direct consequence of pulmonary infection.

Despite being found predominantly in patients with autoimmune PAP, anti-GM-CSF antibodies are also found in the serum of healthy subjects. It is not entirely unusual to observe autoantibodies in healthy individuals and suggests that the progression to disease state involves interactions between specific genes and the environment. In this case, it is possible that autoantibodies directed against GM-CSF function in normal, healthy individuals to prevent excessive inflammatory responses; thus, protecting the lung from damage. Genetically susceptible individuals would have difficulty controlling the amount of anti-GM-CSF antibodies. In this regard, it is possible that autoimmune PAP represents an improper control of anti-GM-CSF antibodies. autoimmune PAP patients may have a genetic link that makes them more susceptible to lung infection but unfortunately, in this study, no link between *HLA* and autoimmune PAP was identified.

## Supporting information

S1 Table PAP patients included in study. Supplemental information for PAP patients including: age, gender, race, ethnicity and GMab levels.
(XLSX)

S2 Table HLA types of PAP patients and 1000 NMDP controls. HLA types of all loci A, B, C, DRB1, DPB1 and DQB1 for patients and controls.
(CSV)

S3 Table HLA-A allele test full results. (CSV)

S4 Table HLA-B allele test full results. (CSV)

S5 Table HLA-C allele test full results. (CSV)

S6 Table HLA-DRB1 allele test full results. (CSV)

S7 Table HLA-DQB1 allele test full results. (CSV)

S8 Table HLA-DPB1 allele test full results. (CSV)

S9 Table HLA-A 1 amino acid association test. (TXT)

S10 Table HLA-A 2 amino acid association test. (TXT)

S11 Table HLA-A 3 amino acid association test. (TXT)

S12 Table HLA-A 4 amino acid association test. (CSV)

S13 Table HLA-B 1 amino acid association test. (TXT)

S14 Table HLA-B 2 amino acid association test. (TXT)

S15 Table HLA-B 3 amino acid association test. (TXT)

S16 Table HLA-B 4 amino acid association test. (CSV)

S17 Table HLA-C 1 amino acid association test. (TXT)

S18 Table HLA-C 2 amino acid association test. (TXT)

S19 Table HLA-C 3 amino acid association test. (TXT)

S20 Table HLA-C 4 amino acid association test. (TXT)

S21 Table HLA-DRB1 1 amino acid association test. (TXT)

S22 Table HLA-DRB1 2 amino acid association test. (TXT)

S23 Table HLA-DRB1 3 amino acid association test. (TXT)

S24 Table HLA-DRB1 4 amino acid association test. (TXT)

S25 Table HLA-DQB1 1 amino acid association test. (TXT)

S26 Table HLA-DQB1 2 amino acid association test. (TXT)

S27 Table HLA-DQB1 3 amino acid association test. (TXT)

S28 Table HLA-DQB1 4 amino acid association test. (TXT)

S29 Table HLA-DPB1 1 amino acid association test. (TXT)

S30 Table HLA-DPB1 2 amino acid association test. (TXT)

S31 Table HLA-DPB1 3 amino acid association test. (TXT)

S32 Table HLA-DPB1 4 amino acid association test. (TXT)

## References

[1] Rosen SH, Castleman B, Liebow AA. Pulmonary alveolar proteinosis. N Engl J Med. 1958;258(23):1123–42. 10.1056/NEJM195806052582301 13552931

[2] Trapnell BC, Whitsett JA, Nakata K. Pulmonary alveolar proteinosis. N Engl J Med. 2003;349(26):2527–39. 10.1056/NEJMra023226 14695413

[3] Goldstein LS, Kavuru MS, Curtis-McCarthy P, Christie HA, Farver C, Stoller JK. Pulmonary alveolar proteinosis: clinical features and outcomes. Chest. 1998;114(5):1357–62. 9824014 10.1378/chest.114.5.1357

[4] Cummings KJ, Virji MA, Park JY, Stanton ML, Edwards NT, Trapnell BC, et al Respirable indium exposures, plasma indium, and respiratory health among indium-tin oxide (ITO) workers. Am J Ind Med. 2016;59(7):522–31. 10.1002/ajim.22585 27219296 PMC4915590

[5] Seymour JF, Presneill JJ. Pulmonary alveolar proteinosis: progress in the first 44 years. Am J Respir Crit Care Med. 2002;166(2):215–35. 10.1164/rccm.2109105 12119235

[6] Trapnell BC. Granulocyte macrophage-colony stimulating factor augmentation therapy in sepsis: is there a role? Am J Respir Crit Care Med. 2002;166(2):129–30. 10.1164/rccm.2205017 12119219

[7] Hercus TR, Thomas D, Guthridge MA, Ekert PG, King-Scott J, Parker MW, et al The granulocyte-macrophage colony-stimulating factor receptor: linking its structure to cell signaling and its role in disease. Blood. 2009;114(7):1289–98. 10.1182/blood-2008-12-164004 19436055 PMC2727416

[8] Dranoff G, Crawford AD, Sadelain M, Ream B, Rashid A, Bronson RT, et al Involvement of granulocyte-macrophage colony-stimulating factor in pulmonary homeostasis. Science. 1994;264(5159):713–6. 8171324 10.1126/science.8171324

[9] Stanley E, Lieschke GJ, Grail D, Metcalf D, Hodgson G, Gall JA, et al Granulocyte/macrophage colony-stimulating factor-deficient mice show no major perturbation of hematopoiesis but develop a characteristic pulmonary pathology. Proc Natl Acad Sci U S A. 1994;91(12):5592–6. 8202532 10.1073/pnas.91.12.5592 PMC44042

[10] Robbins CG, Davis JM, Merritt TA, Amirkhanian JD, Sahgal N, Morin FC 3rd, et al Combined effects of nitric oxide and hyperoxia on surfactant function and pulmonary inflammation. Am J Physiol. 1995;269(4 Pt 1):L545–50. 7485528 10.1152/ajplung.1995.269.4.L545

[11] Suzuki T, Maranda B, Sakagami T, Catellier P, Couture CY, Carey BC, et al Hereditary pulmonary alveolar proteinosis caused by recessive CSF2RB mutations. Eur Respir J. 2011;37(1):201–4. 10.1183/09031936.00090610 21205713

[12] Suzuki T, Sakagami T, Rubin BK, Nogee LM, Wood RE, Zimmerman SL, et al Familial pulmonary alveolar proteinosis caused by mutations in CSF2RA. J Exp Med. 2008;205(12):2703–10. 10.1084/jem.20080990 18955570 PMC2585845

[13] Suzuki T, Sakagami T, Young LR, Carey BC, Wood RE, Luisetti M, et al Hereditary pulmonary alveolar proteinosis: pathogenesis, presentation, diagnosis, and therapy. Am J Respir Crit Care Med. 2010;182(10):1292–304. 10.1164/rccm.201002-0271OC 20622029 PMC3001266

[14] Kitamura T, Tanaka N, Watanabe J, Uchida, Kanegasaki S, Yamada Y, et al Idiopathic pulmonary alveolar proteinosis as an autoimmune disease with neutralizing antibody against granulocyte/macrophage colony-stimulating factor. J Exp Med. 1999;190(6):875–80. 10499925 10.1084/jem.190.6.875 PMC2195627

[15] Sakagami T, Uchida K, Suzuki T, Carey BC, Wood RE, Wert SE, et al Human GM-CSF autoantibodies and reproduction of pulmonary alveolar proteinosis. N Engl J Med. 2009;361(27):2679–81. 10.1056/NEJMc0904077 20042763 PMC4174270

[16] Nepom GT, Erlich H. MHC class-II molecules and autoimmunity. Annu Rev Immunol. 1991;9:493–525. 10.1146/annurev.iy.09.040191.002425 1910687

[17] Nepom GT. Major histocompatibility complex-directed susceptibility to rheumatoid arthritis. Adv Immunol. 1998;68:315–32. 9505093 10.1016/s0065-2776(08)60563-5

[18] Fontenot AP, Maier LA. Genetic susceptibility and immune-mediated destruction in beryllium-induced disease. Trends Immunol. 2005;26:543–9. 10.1016/j.it.2005.08.004 16099719

[19] Uchida K, Nakata K, Trapnell BC, Terakawa T, Hamano E, Mikami A, et al High-affinity autoantibodies specifically eliminate granulocyte-macrophage colony-stimulating factor activity in the lungs of patients with idiopathic pulmonary alveolar proteinosis. Blood. 2004;103(3):1089–98. 10.1182/blood-2003-05-1565 14512323

[20] Benjamini Y, Yekutieli D. Quantitative trait Loci analysis using the false discovery rate. Genetics. 2005;171(2):783–90. 10.1534/genetics.104.036699 15956674 PMC1456787

[21] Freed BM, Schuyler RP, Aubrey MT. Association of the HLA-DRB1 epitope LA(67, 74) with rheumatoid arthritis and citrullinated vimentin binding. Arthritis Rheum. 2011;63(12):3733–9. 10.1002/art.30636 22094856

[22] Mack CL, Anderson KM, Aubrey MT, Rosenthal P, Sokol RJ, Freed BM. Lack of HLA predominance and HLA shared epitopes in biliary Atresia. Springerplus. 2013;2(1):42 10.1186/2193-1801-2-42 23505615 PMC3595468

[23] Roark CL, Anderson KM, Simon LJ, Schuyler RP, Aubrey MT, Freed BM. Multiple HLA epitopes contribute to type 1 diabetes susceptibility. Diabetes. 2014;63(1):323–31. 10.2337/db13-1153 24357703 PMC3868045

[24] Milleron BJ, Costabel U, Teschler H, Ziesche R, Cadranel JL, Matthys H, et al Bronchoalveolar lavage cell data in alveolar proteinosis. Am Rev Respir Dis. 1991;144(6):1330–2. 10.1164/ajrccm/144.6.1330 1741546

